# Autophagy related genes polymorphisms in Parkinson’s Disease; A systematic review of literature

**DOI:** 10.1016/j.prdoa.2025.100312

**Published:** 2025-02-24

**Authors:** Parastoo Yousefi, Shahrzad Ghadirian, Maryam Mobedi, Mehrzad Jafarzadeh, Adib Alirezaei, Ali Gholami, Alireza Tabibzadeh

**Affiliations:** a Department of Virology, School of Medicine, Iran University of Medical Sciences, Tehran, Iran; b Department of Biochemistry and Biophysics, Faculty of Advanced Science and Technology, Tehran Medical Sciences, Islamic Azad University, Tehran, Iran; c Department of Pediatrics Neurology, Arak University of Medical Sciences, Arak, Iran; d Fetal Health Research Center, Hope Generation Foundation, Tehran, Iran; e Endocrine Research Center, Institute of Endocrinology and Metabolism, Iran University of Medical Sciences, Tehran, Iran; f Department of Medical Laboratory, Arak Branch, Islamic Azad University, Arak, Iran; g School of Medicine, Arak University of Medical Sciences, Arak, Iran; h Rajaei Clinical Research Development Unit (CRDU) of Shahid Rajaei Hospital, Alborz University of Medical Sciences, Karaj, Iran

**Keywords:** Parkinson’s disease, Autophagy, Autophagy-related gene, ATG, Single-nucleotide polymorphisms, Variant

## Abstract

**Background:**

Neurodegenerative diseases are mainly a consequence of degenerated proteins in neurons. Parkinson’s disease (PD) is one of the most common neurodegenerative disorders and is characterized by Lewy body deposition. Autophagy is known as one of the cell maintenance mechanisms. Autophagy targets are damaged or degenerated macromolecules and organelles for lysosomal degradation. The role of disrupted autophagy in PD was established earlier. In this regard, the current study aimed to evaluate the frequency and status of the autophagy gene polymorphisms in PD by a systematic review approach.

**Materials and methods:**

In the current study, electronic databases including Scopus, PubMed, and Science Direct were used for the search. The search was performed by using Parkinson’s disease, autophagy, autophagy-related gene, ATG, Single-nucleotide polymorphisms, variant, Sequence variants, and with a date limitation of 2010 to 2023. All original research papers in the English language that evaluate the ATG polymorphisms in PD were included in the study.

**Results:**

The conducted search leads to 2626 primary studies screened based on the inclusion criteria. After the screening stage, 8 studies were included. ATG7 rs1375206 and ATG5 rs510432, rs573775 and rs17587319 were associated with PD. However, some other polymorphisms in ATGs that were not associated with PD were listed.

**Conclusion:**

In conclusion, regardless of the critical role of autophagy in PD pathogenesis, it seems that ATG16 and ATG7 polymorphisms are not associated with PD; however, ATG7 rs1375206 needs more evaluation for a clearer conclusion in future studies. ATG5 and ATG12 polymorphisms seem to be more important in PD. More comprehensive studies about all ATG5, 7, 12, and 16 seem to be urgently required for a conclusive judgment about their role in PD or even other neurodegenerative disorders.

## Highlights

• ATG16 and ATG7 polymorphisms are not associated with PD; however, ATG7 rs1375206 needs more evaluation for a clearer conclusion in future studies.
• ATG5 and ATG12 polymorphisms seem to be more important in PD.
• More comprehensive studies about all ATG5, 7, 12, and 16 seem to be urgently required for a conclusive judgment about their role in PD or even other neurodegenerative disorders.

## 1 Introduction

Neurodegenerative diseases are caused by progressive damage to the nervous tissue of the central nervous system (CNS) and peripheral nervous system (PNS). This damage to the nervous tissue can reduce the quality of life by disabling patients and even could be life-threatening. Some well-established diseases classified as neurodegenerative diseases include Alzheimer's disease (AD), Parkinson's disease (PD), Huntington's disease (HD), and amyotrophic lateral sclerosis (ALS) [1]. The major pathogenesis for all these diseases can be accumulating of damaged or altered proteins such as protein plaque or inclusion bodies in neurons [2], [3]. This pathologic effect is the consequence of insufficient or inefficient protein quality control systems which are meant to eliminate damaged or unnecessary proteins [4].

Parkinson's disease (PD), is one of the important neurodegenerative diseases and its characteristic are dopaminergic neuronal loss and Lewy body deposition in the substantia nigra [5]. PD is considered a common neurodegenerative disease and its incidence increasing each year [6], [7]. Some genes seem to be critical in PD formation. In this regard, the *SNCA* (Synuclein Alpha) gene is assumed to be associated with familial PD. The *SNCA* encodes alpha-synuclein (a-Syn) whose main function is regulating synaptic vesicle trafficking [5], [8]. The a-Syn is considered to be the major component in the Lewy bodies. While other genetic factors associated with PD remain somehow unclear; any alteration in SNCA seems to be associated with PD development [9]. The importance of genetic polymorphisms in PD was determined earlier. Furthermore, the exact clinical significance of these polymorphisms was not clearly understood yet but they potentially could be used for prognostic, diagnostic or therapeutic applications [10]. The role of autophagy seems to be critical in PD pathogenesis; this might be due to the aggregates generated in neuronal cells during PD. One of the cellular mechanisms for overcoming these aggregates in cells is autophagy systems [11].

The autophagy pathway is the critical pathway of cell maintenance and intracellular clearance of degenerated proteins and damaged organelles. Some recent studies suggest that autophagy deficiency is a critical element of PD pathogenesis [12]. Mutations in autophagy lysosomal pathway (ALP) or autophagy regulatory factors are identified as risk factors for PD [13]. Autophagy is a highly evolutionarily conserved cellular pathway for the digestion of damaged macromolecules and organelles through lysosomal degradation. The autophagy can be induced and progressed through macroautophagy, microautophagy, or chaperone-mediated autophagy (CMA). Numerous biological features are tightly associated with autophagy such as development, differentiation of different cells or tissues, adaptation to starvation conditions, quality control of intracellular proteins, tumor suppression, aging, and immune system function, specifically innate immune system [14], [15]. The role of dysregulated autophagy in cardiovascular diseases, diseases based on inflammation, malignancies, or metabolic disorders is well discussed [16], [17]. An association between a-Syn accumulation in PD and alteration in autophagy has been established in previous studies [18], [19]. Regardless of *SNCA*, some other studies also established the importance of autophagy in PD [20], [21].

Autophagy-related genes (ATG) are major components of autophagy regulation. At the current time, more than 30 different *ATG* genes are introduced [14]. Autophagy is a well-regulated process; any alteration in each autophagy regulatory gene can result in a catastrophic pathology [22]. The critical role of *ATG* was established by ATG deletion model studies [16], [23], [24]. The ATG deletion profile in animal models established ATG's importance in malignancy, neurodegenerative diseases, infectious diseases, and metabolic disease development [23], [25], [26]. It seems that genetic variations in *ATG* genes can alter autophagy and might contribute to PD development [27], [28]. The association between some autophagy genes such as microtubule-associated protein 1 light chain 3 beta (*LC-3B*), *ATG5*, and *ATG7*, with sporadic PD is demonstrated by individual studies [29], [30], [31]. The currently available literature about the exact role, status, and frequency of ATG gene polymorphisms in the autophagy system in PD is non-integrated, and there seems to be a gap of knowledge about this gene polymorphism status in PD is undeniable. In this regard, the current study aimed to evaluate the frequency and status of the autophagy gene polymorphisms in PD by a systematic review approach.

## 2 Materials and methods

### 2.1 Search strategy and inclusion criteria

All study procedures are established based on the PRISMA criteria (30). In the current study, electronic databases including Scopus, PubMed, and Science Direct were used for the search. Google Scholar was used to search to obtain any possible relevant grey literature. The search was performed using Parkinson's disease, autophagy, autophagy-related gene, ATG, Single-nucleotide polymorphisms, variant, and Sequence variants as keywords and with a date limitation of 2010 to December 7th, 2023. The exact search queries are provided in Supplementary Data 1.

All original research papers in the English language that evaluate the ATG polymorphisms or polymorphisms for any core gene in autophagy in PD were included in the study. All review articles, papers with non-relevance topics, and papers with a high risk of bias were excluded from the final systematic review. The Core genes in autophagy in this study are mentioned to all core genes of classical degradative autophagy (initiation: *ULK1*, *ULK2*, *FIP200*, and *ATG13*, nucleation: *VPS34*, *VPS15*, *BECN1*, and *ATG14*, and *ATG9*, expansion and elongation: *ATG3*, 5, 7, 12, 16, *LC-3*, fusion: *LC-3*, HOPS complex, *RAB7*) [32].

This review was not registered. In the first step, all search results listed in the EndNote 20 (Thomson Reuters, Canada) and screened based on title, and then screening was performed based on the abstract. All relevant studies were used for full-text retrieval. All included studies were from the mentioned databases, all studies evaluated the ATG polymorphisms in PD.

### 2.2 Screening and data extraction

All data from the search was listed in the EndNote 20 (Thomson Reuters, Canada). The screening performed by two independent authors and a third expert author strategy used for matters of disagreement. Based on the inclusion criteria, included studies used for data extraction. Extracted data included the Author's name, Country, Year of the conducted study, study population, evaluated gene, and evaluated polymorphism frequency in patients and control groups.

### 2.3 Study bias assessment

The Newcastle-Ottawa Scale (NOS) checklist was used for bias assessment in the included studies. The checklist included Representativeness of the Exposed Cohort, Selection of the Non-Exposed Cohort, Ascertainment of Exposure, and Outcome of Interest by using a star scoring system [33].

## 3 Results

### 3.1 Search results and bias assessment

**Fig. 1:**
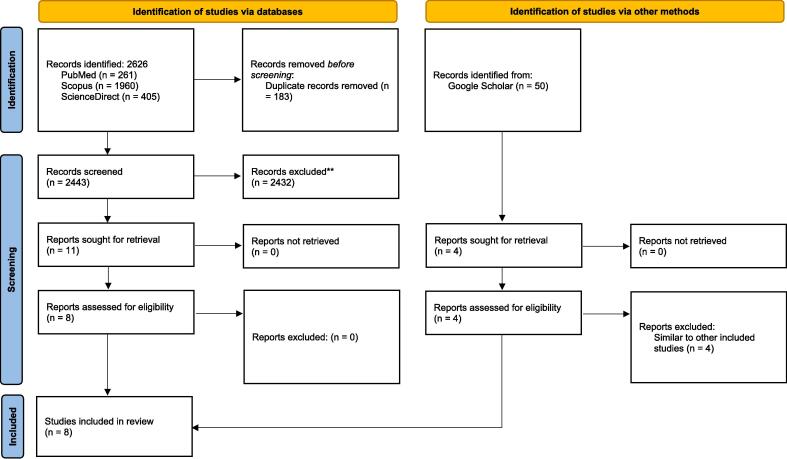

The conducted search leads to 2626 primary studies. The conducted studies are screened based on the inclusion criteria. After the screening stage 8 studies were included. The study search diagram is presented in Fig. 1. The risk of bias assessment represents acceptable results in all of the included studies; more details about the risk of bias assessment are provided in Supplementary Data 2.

### 3.2 ATG polymorphisms and PD

In the current study, we include 8 studies about the ATG polymorphisms and PD. The majority of included studies were in case-control settings. Most included studies are relevant to China and one is from Spain. The *ATG5* (2 studies), *ATG7* (3 studies), *ATG12* (1 study), and *ATG16* (2 studies) were evaluated. A summary of included studies is listed in Table 1. The *ATG12* rs26538 C > T can alter the transcription factor binding; however, this polymorphism was not associated with PD. On the other hand, the *ATG7* rs1375206 C > A, G, T was associated with susceptibility to late-onset sporadic PD. Meanwhile, other *ATG7* evaluated polymorphisms (11313215 A > G, 11,313,910 G > A, rs7625184 T > C, rs2606750 T > C) did not show any association with PD. Some of the ATG5 polymorphisms (rs510432 A > G, rs573775 C > T, rs17587319 C > G,) represent association with PD and are also suggested as prognostic markers. Meanwhile, rs510432 A > G did not show any association. All of the *ATG16L1* evaluated polymorphisms did not represent any statistically significant association with PD (rs146693112 G > A, rs1816753 T > C, rs12476635 T > C, rs74599577 A > T, rs777177003). A summary of all evaluated polymorphisms in 8 included studies are listed in Table 2.

**Table 1:** a summary of included studies of ATG polymorphisms in PD patients.

Study features			Population		Evaluated polymorphism						Main conclusion	Ref.
Author	Country	Year	PD	HC	gene	polymorphism	Polymorphisms in patients		Polymorphisms in control			
							Heterozygote	homozygote	Heterozygote	homozygote		
Li	China	2017	136	133	*ATG12*	rs26538 C > T	58	22	73	13	Prevalence of rs26538 did not represents any statistical difference between groups Alterations in ATG12 promoter effect the transcription factors binding	[21]
Zhao	China	2020	124	105	*ATG7*	rs1375206 C > A, G, T	72	8	63	2	rs1375206 polymorphism in *ATG7* may not be associated with late-onset sporadic PD A-T haplotype may be associated with susceptibility to late-onset sporadic PD Plasma ATG7 protein levels were significantly higher in PD compare with control	[34]
Chen	China	2012	101	148	*ATG7*	11,313,215 A > G	42	14	68	11	Prevalence of both variants did not represents any statistical difference between groups Four novel heterozygous variants were identified in five PD patients, that decrease transcriptional activity	[35]
						11,313,910 G > A	49	33	72	56		
Zou	China	2022	312	309	*ATG7*	rs7625184 T > C	132	31	127	29	No association between ATG7 and PD	[36]
						rs2606750 T > C	169	89	162	104		
Han	China	2022	120	100	*ATG5*	rs510432 A > G	52	46	42	38	ATG5 polymorphism and protein low levels are associated with susceptibility to PD and with cognitive impairment ATG5 as biomarker to assess the severity and prognosis of PD	[37]
						rs573775 C > T	72	16	52	22		
						rs17587319 C > G	16	4	40	8		
Chen	China	2013	100	139	*ATG5*	rs510432 A > G	53	30	65	41	novel heterozygous variant, 106774459 T > A, in one female PD patient 106774459 T > A, significantly enhanced the transcriptional activities of ATG5 rs510432 frequency did not show any statistical significance in PD	[38]
Wang	China	2017	151	174	*ATG16L1*	rs146693112 G > A	2	0	3	0	genetic variants of ATG16L1 promoter may not be a risk factor for sporadic PD	[39]
						rs1816753 T > C	37	0	46	0		
						rs12476635 T > C	132	0	141	0		
						rs74599577 A > T	9	0	7	0		
Gomez-Martín	Spain	2023	73	37	*ATG16L1*	rs777177003	1	0	0	0	A rare 8 bp insertion in ATG16L1 promotor	[40]

PD: Parkinson’s disease, HC: Healthy controls,

**Table 2:** A summary of evaluated polymorphisms and ATGs in 8 included studies.

Evaluated polymorphisms by 8 distinct studies [21], [34], [35], [36], [37], [38], [39], [40]							
Associated with PD				Did not represent association with PD			
Gene	polymorphisms	Prevalence range		Gene	polymorphisms	Prevalence range	
		PD	HC			PD	HC
*ATG7*	rs1375206 C > A, G, T*	65 %**	61 %	*ATG12*	rs26538*** C > T	58 %	64 %
*ATG5*	rs510432*** A > G	81–83 %	76–80 %	*ATG7*	rs11313215*** A > G	55 %	53 %
					rs11313910*** G > A	87 %	86 %
					rs7625184*** T > C	52 %	50 %
	rs573775 C > T	73 %	74 %		rs2606750*** T > C	82 %	54 %
	rs17587319 C > G	16 %	48 %	*ATG5*	rs510432*** A > G	81–83 %	76–80 %
				*ATG16*	rs146693112 G > A	1.3 %	1.7 %
					rs1816753 T > C	24 %	26 %
					rs12476635 T > C	87 %	81 %
					rs74599577 A > T	6 %	4 %
					rs777177003	1.3 %	0 %

* Not significant in genotype distribution but haplotype A-T was significantly related to late-onset sporadic PD.

** Data obtained in one study and needs more investigation for more clarification.

*** Data from two independent study seems to be in conflict.

## 4 Discussion

**Fig. 2:**
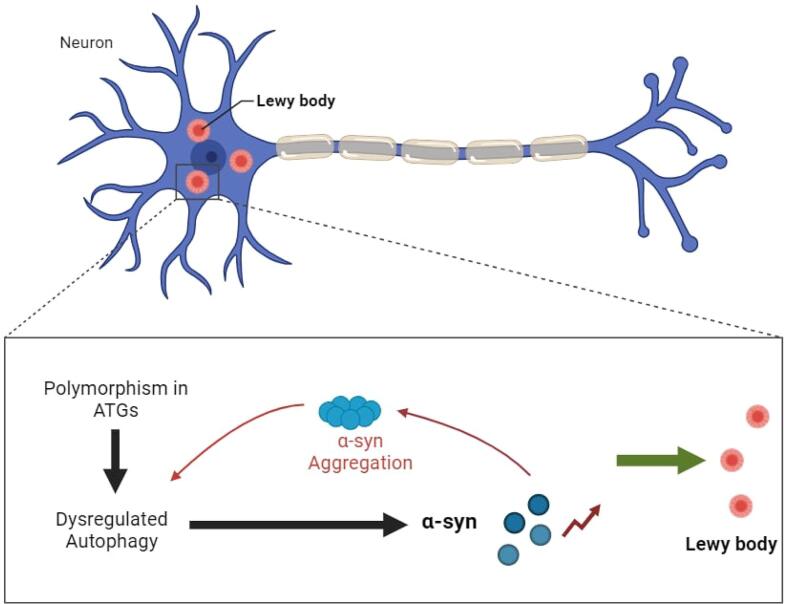
Polymorphism in ATGs results in impaired autophagy then the autophagy will not be able to clear some aggregates in cells such as a-syn. This a-syn will be a cause for Lewy body generation and PD development or progression.

The association between neurodegenerative disorders and autophagy is established by a variety of studies. Impaired autophagy maturation could be a clue about the pathogenesis of neurodegenerative diseases. In this regard, the mutation is a wide variety of genes that seem to be associated with the pathogenesis mechanisms. The most important impaired gene in PD is the *SNCA* [9] and some ATGs [21], [34]. Some other mutations in autophagy genes regardless of ATGs assumed to be associated with autosomal recessive or sporadic juvenile-onset Parkinson's disease; for instance, we can mention *PARK2/Parkin* and *PARK6/PINK1* [41], [42], [43]. As a conclusive perspective, polymorphism in ATGs results in impaired autophagy. This impaired autophagy will not be able to clear some aggregates or damaged materials in cells such as a-syn. This a-syn will be a cause for Lewy body generation and PD development or progression (Fig. 2).

The majority of PD cases are sporadic and disease etiology is still not completely understood. However, the interaction between genetic and environmental factors is considered the main cause of PD development [44]. Autophagy and lysosomal imbalance have been discussed in different neurodegenerative disorders [45]. The genetic variations in autophagy genes, especially ATGs can alter the autophagy function [39]. In this regard, the current study aimed to evaluate all relevant studies about the frequency and function of the ATG gene polymorphisms in PD based on a systematic review approach. The results of the current study by considering 8 individual primary studies indicate that *ATG7* rs1375206 and *ATG5* rs510432, rs573775, and rs17587319 were associated with PD. However, another polymorphism was not associated with PD; including *ATG12* rs26538, ATG7 (rs11313215, rs11313910, rs7625184 and rs2606750), *ATG5* rs510432 and *ATG16L1* (rs146693112 and rs1816753).

Zhao et al. showed that the rs1375206 polymorphism of *ATG7* was not significant in genotype distribution between healthy controls and PD patients but haplotype A-T was significantly related to late-onset sporadic PD. Also, the *ATG7* levels are statistically significant differences between PD and controls [34]. The ATG7 polymorphisms in the coding region seem to be associated with Huntington’s disease [46], while other studies about evaluated polymorphisms in *ATG7* are in the same way about not association of this polymorphism with PD. In this regard, Chen et al. indicate that more frequent *ATG7* promoter polymorphisms (11313215 A > G and 11,313,910 G > A) did not show any statistically significant difference between sporadic PD patients and ethnic-matched healthy controls. Meanwhile, Chen et al. report four novel heterozygous variants in five PD patients only and not the controls [35]. Similar results about not a statistically significant difference in prevalence of *ATG7* rs7625184 T > C and rs2606750 T > C in PD were reported by Zou et al. [36]. In conclusion, it seems that these evaluated polymorphisms in *ATG7* are not associated with PD however, *ATG7* rs1375206 needs more evaluation for a clearer conclusion in future studies.

The conducted studies about *ATG12* and diseases are limited. The role of *ATG12* expression in spinocerebellar ataxia type 7 [47], [48] is introduced earlier. In cell line models *ATG12* over-expression can inhibit autophagosome formation [49]. There are limited primary studies about *ATG12* and PD. In the only conducted study in this field, Li et al. found five novel heterozygous polymorphisms in sporadic PD patients which can alter the transcriptional activity of *ATG12*. However, the *ATG12* rs26538 C > T did not represent any statistically significant difference between healthy and PD groups [21]. More comprehensive studies about *ATG12* seem to be urgently required for a conclusive judgment about its role in PD or even other neurodegenerative disorders.

Chen and colleagues represent a novel polymorphism in only one PD patient in the ATG5 promotor (106774459 T > A) with increased transcriptional activities of the *ATG5* promoter. Furthermore, Chen's study showed that *ATG5* rs510432 A > G was not associated with PD [38]. In contrast, a conducted study by Han et al. represents a significant correlation between *ATG5* rs17587319 with PD and its prognosis in cognitive impairment [37]. It seems to be, *ATG5* polymorphisms are more important in PD. These studies provide a view about the future evaluation of ATG5 in PD in expression format or other polymorphisms.

The same results about the *ATG16* polymorphisms and PD were reported by two distinct studies [39], [40]. All evaluated ATG16L1 polymorphisms with Wang et al. (rs146693112 G > A, rs1816753 T > C, rs12476635 T > C and rs74599577 A > T) and Gomez et al. (rs777177003) could show any association with PD. These findings recommend that *ATG16L1* does not seem to be associated with PD; at least none of the evaluated polymorphisms.

Another important aspect of differences between these studies is methodological and demographic differences. The majority of these studies used the same methodology with slight differences in polymorphism detection and study setting [21], [34], [35], [36], [37], [38], [39], [40]. The demographical data about age, sex, and geographical distribution of included patients in primary studies indicate some differences which could be a confounding factor, especially about the geographical distribution of studies. The majority of studies focused on the Chinese population [21], [34], [35], [36], [37], [38], [39], [40].

Evaluation of polymorphisms in different genes is determined as a tool to provide more understanding of diseases. These polymorphisms in different genes are associated with a variety of infections or non-infectious diseases [50], [51], [52]. Understanding these polymorphisms can provide insight into the disease pathogenesis through the lens of molecular or gene interaction [53], [54]. Since the autophagy is a central mechanism in cell maintenance and the cell maintenance is a critical issue in PD assessment of polymorphisms in these genes could be useful in better understanding of disease which provides a clue about further research. Polymorphisms could also provide diagnostic or prognostic tools in PD [55]. Further researches could be focused on treatment responses or the prognostic role of some more frequent mutations (Such as *ATG5* or *ATG7*) in PD due find more effective treatment and move into personalized medicine and target therapy area for PD [56]. Also, this kind of more frequent polymorphism in PD could be a possible diagnostic tool after confirmation in large-scale studies. Using polymorphisms as a diagnostic tool is helpful to provide a more precise diagnosis as a more reliable test [57].

Another aspect of polymorphism evaluation for PD is obtained data from Genome-wide association studies (GWAS) [58]. Different studies focused on GWAS data from polymorphisms in PD; in this regard, a conducted study by Nalls et al. [59] indicates 90 variants in different genes and loci that explain 16–36 % heritable risk of PD. In the Nalls et al. no core autophagy genes were reported while some autophagy related genes were represented to be associated with P risk including Mitogen-activated protein 4 kinase (*MAP4K*) and Microtubule-associated protein tau (*MAPT*). Similar findings were also reported by Leonard and colleagues. The only autophagy related gene that Leonard et al. reported were *MAPT* while some other critical genes and its possible effects on trials were discussed [60]. Another study by Kim et al. [61] evaluates the GWAS data and preform a *meta*-analysis. Regarding some critical genes such as Metal Response Element Binding Transcription Factor 2 (*MTF2*), Phosphatidylinositol-4,5-Bisphosphate 3-Kinase Catalytic Subunit Alpha (*PIK3CA*), Adducin 1 (*ADD1*) no close gene to autophagy pathway were reported except Ras-associated binding 7 (*Rab7*). Verity of studies evaluates other genes and represents polymorphisms association with PD in different clinical conditions. For instance, glucocerebrosidase (*GBA*) rs12726330 and PD age onset [62]. By considering GWAS and other studies about polymorphisms, it seems the autophagy gene polymorphism in PD is at the beginning of its way and needs more comprehensive primary studies to reach a clear conclusion.

The major limitation of our current study was due to a limited number of primary studies. Another important aspect is the geographical distribution of included studies. Except for Gomez et al., all included studies evaluated the Chinese population [21], [34], [35], [36], [37], [38], [39], [40]. More studies in the field of different ATG polymorphisms and PD in different geographical locations seem to be urgent for the conclusion about the role of these polymorphisms in ATG expression, autophagy system function, and PD development, diagnosis, or prognosis. For each genetic locus and polymorphism different genetic models, such as dominant, recessive, or co-dominant models could be recorded. The heterozygote and homozygote comparison of the PD and HC groups in Table 1 is just a preliminary analytical method, and is not sufficient to determine genetic risk. Further analysis should consider different genetic models and evaluate the relationship between genotype and disease under these models. Some of the other limitations include Geographical Diversity, Small sample size, Lack of Functional Analysis, Potential Confounding Factors, or bias and heterogeneity in PD Diagnosis. Limited Geographical Diversity (majority of included studies predominantly focused on populations from China) This limitation may restrict the generalizability of the findings to other populations with different genetic backgrounds or environmental exposure factors. Some of the included studies had relatively small sample sizes, which could affect the precision of the results. Studies with larger sample sizes would provide more robust estimates of the associations between ATG polymorphisms and Parkinson's disease. The included studies varied in their study designs, with differences in case-control settings and methodologies. The study primarily focused on evaluating the frequency of ATG gene polymorphisms in PD but did not include functional analyses to elucidate the underlying biological mechanisms. Future studies incorporating functional analyses could provide deeper insights into the functional consequences of these polymorphisms. The included studies may not have fully accounted for potential confounding factors, such as age, sex, lifestyle factors, or comorbidities, which could influence the associations between ATG polymorphisms and PD. Future studies should consider adjusting for these confounders to improve the accuracy of the results. There may be a risk of publication bias, as the included studies were limited to those published in English-language journals and indexed in specific electronic databases. Studies with null or nonsignificant findings may be less likely to be published, leading to an overrepresentation of positive associations in the literature. The cross-sectional nature of some included studies limits the ability to establish temporal relationships between ATG gene polymorphisms and PD. Longitudinal studies with repeated measures over time would provide more robust evidence of causal relationships. There may be heterogeneity in the diagnosis and classification of PD across the included studies, which could influence the consistency and comparability of the results. Standardizing diagnostic criteria or conducting sensitivity analyses based on disease subtypes could address this limitation. We tried our best to provide a good overview, however, future new and more narrow systematic reviews are essential for more clear understanding of this field. This also needs to be kept in mind, the primary studies and our secondary study are still premature and this manuscript tried to highlight the gaps and leads for future studies and provoke researchers to focus on this field for a clear conclusion in the future.

## 5 Conclusion

In conclusion, regardless of the critical role of autophagy in PD pathogenesis, it seems that *ATG16* and *ATG7* polymorphisms are not associated with PD; however, *ATG7* rs1375206 needs more evaluation for a clearer conclusion in future studies. *ATG5* and *ATG12* polymorphisms seem to be more important in PD. More comprehensive studies about all *ATG5*, 7, 12, and 16 seem to be urgently required for a conclusive judgment about their role in PD or even other neurodegenerative disorders. The autophagy gene polymorphisms and expression could be a key to more understanding of PD pathogenesis and seem to be a lead for future research. More understanding of the role of autophagy in PD could be a promising element in clinical practice for PD.

## 6 Ethics statement

Current study did not include any primary clinical, biomedical, study participants or any new clinical data and all included data obtained from available scientific databases.

## Authors contribution

The concept and design by SG, PY, AT search and screening documents by SG, PY, MJ, AG, AA, MB the manuscript preparation and final revision of manuscript AT, PY, AA, AG and MJ.

## CRediT authorship contribution statement

**Parastoo Yousefi:** Writing – review & editing, Writing – original draft, Meth, odology, Data curation, Conceptualization. **Shahrzad Ghadirian:** Writing – review & editing, Writing – original draft, Investigation, Data curation. **Maryam Mobedi:** Writing – review & editing, Writing – original draft, Investigation, Data curation. **Mehrzad Jafarzadeh:** Writing – review & editing, Writing – original draft, Investigation, Data curation. **Adib Alirezaei:** Writing – review & editing, Writing – original draft, Investigation. **Ali Gholami:** Writing – review & editing, Writing – original draft, Investigation. **Alireza Tabibzadeh:** Writing – review & editing, Writing – original draft, Supervision, Methodology, Investigation, Data curation, Conceptualization.

## Declaration of competing interest

The authors declare that they have no known competing financial interests or personal relationships that could have appeared to influence the work reported in this paper.
