# Supplementary material for: Autophagy related genes polymorphisms in Parkinson’s Disease; A systematic review of literature
Source: Clin Park Relat Disord. 2025 Feb 24;12:100312. doi: 10.1016/j.prdoa.2025.100312 (PMC11910361; doi:10.1016/j.prdoa.2025.100312)
Supplement: Supplementary Data 1 [file mmc1.docx]

**Supplementary data 1**

Search strategy:

Range: 2010- December 7^th^ 2023

| Database | Query | Number |
| --- | --- | --- |
| PubMed MESH | ((("Parkinson Disease"[Mesh]) AND "Autophagy"[Mesh]) AND "Polymorphism, Single Nucleotide"[Mesh]) | 3 |
| PubMed | "Parkinson Disease"[tiab]) AND ("Autophagy"[tiab]) OR "ATG" OR "autophagy-related gene") AND ("Polymorphism, Single Nucleotide"[tiab] OR "Polymorphism") | 258 |
| Scopus 1 | “Parkinson’s disease” AND ( autophagy OR "ATG" OR "autophagy-related gene" ) AND ( "Polymorphism" OR single AND nucleotide AND polymorphism OR mutation ) | 7 |
| Scopus 2 | "Parkinson's disease" AND autophagy AND polymorphism | 39 |
| Scopus 3 | "Parkinson's disease" AND autophagy | 1914 |
| ScienceDirect | "Parkinson's disease" AND autophagy AND polymorphism | 405 |

**Supplementary data 2**

Risk of bias assessment by Newcastle-Ottawa Scale (NOS) checklist:

| Author | Ref. | Selection | | | | Comparability | Outcome |
| --- | --- | --- | --- | --- | --- | --- | --- |
|  |  | Representativeness of the Exposed Cohort | Selection of the Non-Exposed Cohort | Ascertainment of Exposure | Outcome of Interest Was Not Present at Start of Study |  |  |
| Li | (19) | 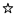 | 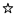 | 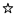 | 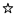 | 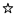 | 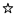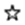 |
| Zhao | (32) | 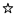 | 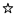 | 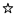 | 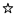 | 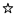 | 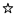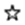 |
| Chen | (33) | 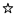 | 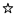 | 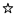 | 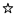 | 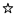 | 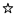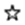 |
| Zou | (34) | 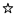 | 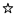 | 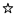 | 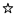 | 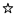 | 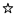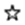 |
| Han | (35) | 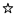 | 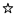 | 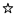 | 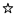 | 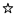 | 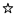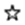 |
| Chen | (36) | 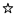 | 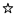 | 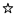 | 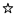 | 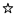 | 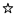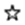 |
| Wang | (37) | 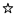 | 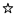 | 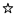 | 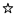 | 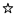 | 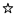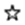 |
| Gomez-Martín | (38) | 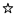 | 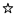 | 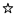 | 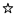 | 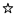 | 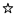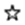 |
